# Treatment outcomes of individuals with tuberculosis identified from mobile chest X-ray–based active case finding in Pakistan

**DOI:** 10.5588/pha.26.0030

**Published:** 2026-08-24

**Authors:** S.M.A. Zaidi, K.C. Horton, H. Esmail

**Affiliations:** 1 WHO Collaborating Centre for Tuberculosis Research and Innovation, UCL Centre for Global Tuberculosis Research, Institute for Global Health, University College London, London, UK;; 2 Community Health Solutions, Karachi, Pakistan;; 3 Department of Infectious Disease Epidemiology, London School of Hygiene and Tropical Medicine, London, UK;; 4 MRC Clinical Trials Unit at University College London, London, UK.

**Keywords:** tuberculosis, diagnosis, radiography, asymptomatic TB, CAD, lost-to-follow-up

## Abstract

Mobile X-ray–based active case finding is being scaled up across high-burden countries to improve detection of early and asymptomatic tuberculosis (TB). However, limited evidence is available for treatment outcomes in programmatic settings. We investigated outcomes of 1,023,488 individuals screened through community-based mobile X-rays in Pakistan. Among 7,381 individuals treated for TB, 4,104 (55.6%) did not have bacteriological confirmation suggesting low bacterial disease burden. Individuals with bacteriologically unconfirmed TB were more likely to be lost to follow-up relative to those with bacteriologically confirmed TB (18.3% vs. 13.2%), highlighting the need for strategies to ensure individuals remain engaged in care throughout treatment.

An estimated 10.7 million people (95% uncertainty interval [UI]: 9.9–11.5 million) fell ill with tuberculosis (TB) in 2024, leading to 1.23 million (95% UI: 1.13–1.33 million) deaths.^1^ Among incident cases, 8.3 million individuals were diagnosed, among whom only 36% of those with pulmonary TB were bacteriologically confirmed.^1^ Active case finding (ACF) is a case-detection strategy, defined as screening for TB outside of health facilities, and is being increasingly used by TB programmes to improve case-detection.^2^ ACF has also gained prominence with emerging evidence that a significant proportion of individuals with TB may not report any symptoms and that people may be identified earlier through chest X-ray (CXR)-based screening supported by computer-aided diagnostics (CAD) software. In 2024, the World Health Organization updated its case-definitions to include the terms asymptomatic TB, as well as bacteriologically confirmed and bacteriologically unconfirmed TB for individuals identified through screening.^3^ Evidence from historical studies suggests that individuals with CXR suggestive of TB but bacteriologically unconfirmed disease progress to positive TB at rate of 10% per year without treatment.^4^

However, case-detection represents only one part of the care spectrum, and it is important to ensure that individuals identified during screening are also successfully treated. The goal of ACF is not simply case-detection but ensuring that future morbidity or mortality is prevented at an individual level and that transmission is interrupted at the community level. Limited contemporary evidence is available for treatment outcomes of individuals identified through programmatic screening. Pakistan was an early adopter of CAD–CXR screening and we recently published a large retrospective analysis of programmatic data of case-detection yields.^5^ Given the scale-up of ACF and increased interest in the topic, we further report on the diagnostic work-up and treatment outcomes of individuals diagnosed through community screening.

## METHODS

This was a descriptive, retrospective analysis of programmatic data.

### Community screening procedures

Screening procedures have been previously described in detail.^5^ Briefly, community-based screening events are called ‘camps’ in Pakistan. These involved a single day’s activity during which a mobile van equipped with digital CXR and CAD software was parked at the intended site and the nearby community was motivated to undergo voluntary screening for TB. Individuals with a CXR suggestive of TB or those with symptoms were encouraged to expectorate a spot sputum specimen. Individuals diagnosed with TB received medication primarily at purpose-built TB diagnostic and treatment centres in the private sector (*Sehatmand Zindagi Centers*), or at other private or public-sector TB facilities.^6^

### Outcome definitions

Bacteriologically confirmed TB included individuals who were initiated on treatment after testing positive on a bacteriological test (Xpert MTB/RIF or less frequently using smear-microscopy). Bacteriologically unconfirmed TB included individuals who were initiated on treatment with radiological or clinical features suggestive of TB but were either negative on a bacteriological test or were unable to expectorate a sputum sample. Treatment success included individuals who had documented evidence of monthly drug-collection visits for the full duration of TB treatment (treatment completed) as well as those who additionally had negative smear-microscopy results during follow-up (treatment cure). Lost to follow-up included individuals who did not have documented evidence for drug-collection visits for the full duration of TB treatment.

### Data analysis

TB notifications and outcomes data were utilised for this analysis for cases diagnosed from 2017 to 2020. Cases diagnosed after April 2020 were excluded from this analysis due to lockdowns associated with COVID-19 that led to challenges in ensuring treatment adherence and therefore may bias reporting outcomes.^7^ Differences in treatment outcomes between individuals with bacteriologically confirmed and unconfirmed were investigated for statistical significance using χ² tests.

## RESULTS

A total of 7,381 individuals were initiated on treatment for TB during the study period from 1,023,488 screened. Among those initiated on treatment, 3,277 (44.4%) were bacteriologically confirmed and 4,104 (55.6%) were bacteriologically unconfirmed (see Figure). Among individuals treated with bacteriologically unconfirmed TB, 2,127 (51.8%) had negative Xpert results, 1,808 (44.1%) did not expectorate sufficient sputum for Xpert testing, and 169 (7.3%) had invalid test results. Overall, 75% (n = 5,534/7,381) of individuals identified through screening were treated successfully and this proportion was slightly higher among individuals with bacteriologically confirmed TB (76.7%) relative to those with bacteriologically unconfirmed TB (73.6%) (*P* value <0.01) (see Table). A higher proportion of individuals with bacteriologically unconfirmed TB (18.3%) were lost to follow-up, relative to those with bacteriologically confirmed TB (13.2%) (*P* value <0.01). Overall, 265 individuals (3.6%) died during treatment and this proportion was similar among both groups (*P* value 0.93). A total of 17 (0.5%) of individuals with bacteriologically confirmed TB and 2 (0.05%) individuals with bacteriologically unconfirmed TB failed treatment.

**FIGURE.**
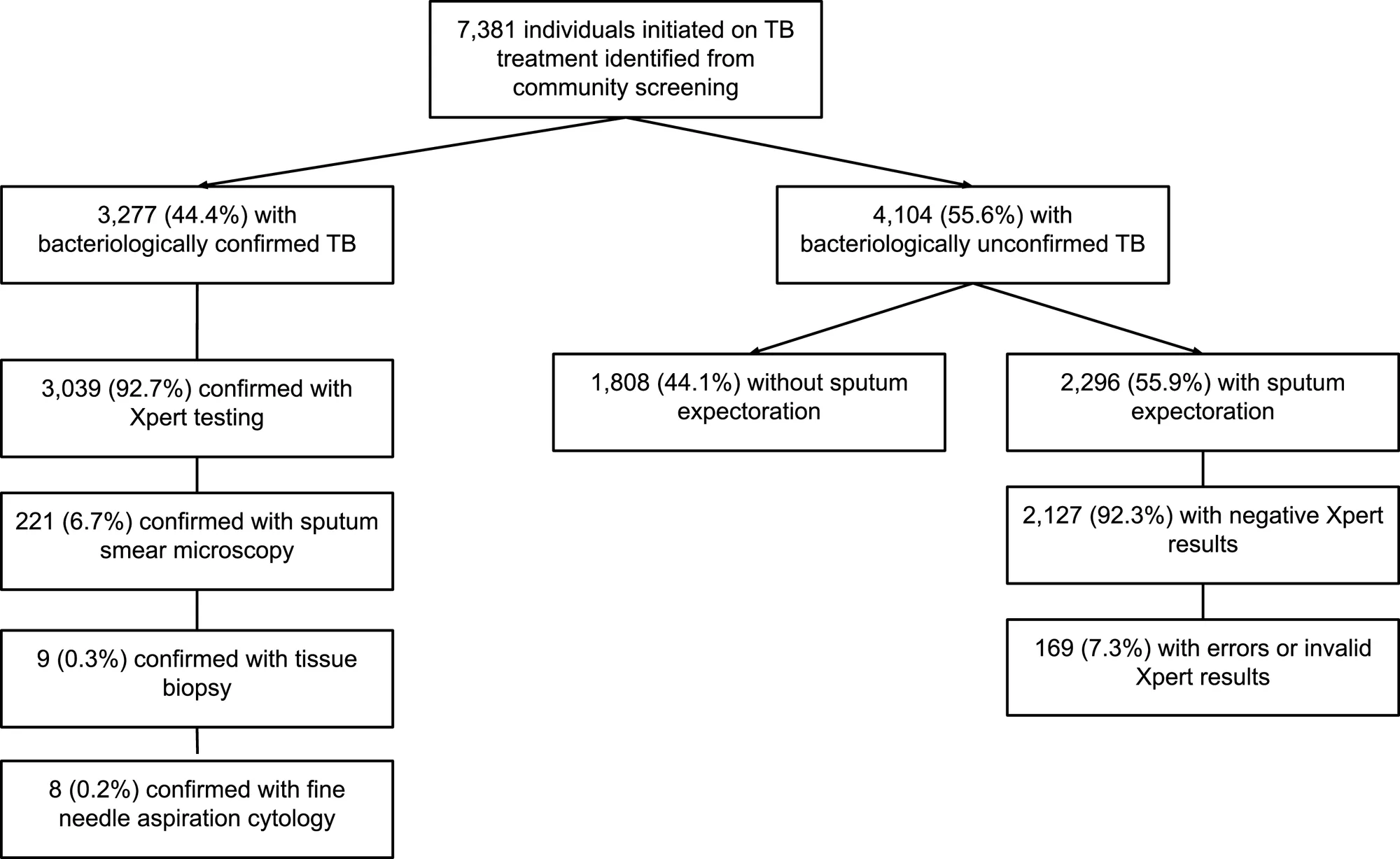
Diagnostic work-up of individuals initiated on tuberculosis (TB) treatment identified from mobile X-ray–based active case finding in Pakistan.

**TABLE.** Treatment outcomes of individuals initiated on tuberculosis (TB) treatment identified from mobile X-ray–based active case finding in Pakistan.

	Bacteriologically confirmed TB		Bacteriologically unconfirmed TB		Total		*P* value^A^
Treatment success (cured or completed)	2,513	76.7%	3,021	73.6%	5,534	75.0%	<0.01
Lost to follow-up	432	13.2%	749	18.3%	1,181	16.0%	<0.01
Initiated on DR-TB treatment	80	2.4%	9	0.2%	89	1.2%	<0.01
Transferred out to DS-TB site	118	3.6%	175	4.3%	293	4.0%	0.93
Died	117	3.6%	148	3.6%	265	3.6%	0.15
Treatment failure	17	0.5%	2	0.0%	19	0.3%	<0.01
	3,277		4,104		7,381		

DR-TB = drug-resistant TB; DS-TB = drug-sensitive TB.

A Significance analysed through χ² tests.

### Lessons learned

This is the largest reported analysis of diagnostics and treatment outcomes of mobile CXR supported ACF and provides two key lessons for TB programmes. First, over 50% of individuals with bacteriologically unconfirmed TB had negative Xpert results. This suggests that despite a negative confirmatory diagnostic, these individuals had sufficient radiological findings or symptomatology for clinicians to initiate TB treatment. It is likely that these individuals had a low bacillary burden that was not detected even with molecular diagnostics. Programmes will need to develop appropriate clinical management guidelines to ensure that the most appropriate care is provided.

Second, the overall treatment success rate (75%) for individuals identified was lower than the country average of 92% and lost to follow-up was higher among those with bacteriologically unconfirmed TB. It is possible that individuals identified during screening were less sick or were asymptomatic and therefore did not feel the need to continue TB treatment, particularly those with bacteriologically unconfirmed TB. A positive confirmatory diagnostic result may have influenced patient behaviour by increasing motivation to continue taking medication. While screening was conducted close to patients’ homes, follow-up still required travel to TB centres and this may have been a barrier to care, particularly for women.^8^ Programmes need to consider appropriate linkages between screening teams and treatment centres as well as provide individuals under treatment with counselling and adherence support.

This study has some limitations. It is likely that individuals with bacteriologically unconfirmed TB may have been identified using the newer Xpert Ultra cartridge as ‘Trace’ results.^9^ Conversely, it is also possible that these individuals would have self-cured without treatment.^10^ Additional research is required to develop more sensitive diagnostic assays, and prospective cohorts will be required to determine the risk of progression for this clinical phenotype. This study was in the programmatic setting, and treatment completion was based on documented follow-up clinic visits and not using directly observed therapy.
